# Health before pregnancy and eligibility for parental leave benefits: a Swedish total population cohort study

**DOI:** 10.1186/s12889-025-22248-8

**Published:** 2025-03-18

**Authors:** Amy Heshmati, Andrea Dunlavy, Eleonora Mussino, Sara Fritzell, Sol P. Juárez

**Affiliations:** 1 Centre for Health Equity Studies (CHESS), Stockholm University/Karolinska Institutet, Stockholm, Sweden; 2 Department of Public Health Sciences, Stockholm University, Albanovägen 12, Stockholm, 10 691 Sweden; 3 Department of Global Public Health, Karolinska Institutet, Stockholm, Sweden; 4 Stockholm University Demography Unit (SUDA), Stockholm, Sweden

**Keywords:** Health in All Policies, Health inequalities, Mental health, Parental leave

## Abstract

**Background:**

Parental leave generosity is protective for mothers’ mental health in the postpartum period and beyond. Strong work requirements exist for parents in Sweden to receive more generous benefits which might penalise individuals who, due to poor health, have a weak labour market attachment. The aim of the study was to examine whether mothers with poor health prior to pregnancy are less likely to be eligible for more generous benefits in Sweden.

**Methods:**

We used total population registers to study first-time mothers, aged 25–45 years, who were resident in Sweden and gave birth between 1 January 2009 and 30 September 2013 (*n* = 151,452). We used logistic regression to examine the association between health one and two consecutive years prior to pregnancy (to assess chronicity) and eligibility for earnings-related parental leave benefits.

**Results:**

Mothers who were admitted to hospital or received specialist outpatient care for any health condition in the year prior to pregnancy were less likely to be eligible for earnings-related benefits (OR 0·79, 95%CI 0·76–0·83) compared to healthy mothers, particularly those with chronic health issues (OR 0·64, 95%CI 0·62–0·68). Findings were driven by mothers with mental disorders (OR 0·22, 95%CI 0·20–0·23 for the year before pregnancy), and associations were stronger for those with chronic health issues and for Swedish-born mothers.

**Conclusion:**

Mothers with prior health conditions, particularly mental disorders, are less likely to benefit from the protective health effect of parental leave as they may not meet the eligibility requirements for more generous remunerations. This study highlights how the strong work requirement for accessing generous parental leave benefits could unintentionally exacerbate socioeconomic inequalities between mothers with and without poor mental health. Easing work requirements for eligibility to more generous parental leave remuneration could help reduce these inequalities and thus promote better mental health for all, particularly among more disadvantaged groups. As such, our findings empirically support the need for adopting the *Health in All Policies* framework when designing parental leave policies in order to minimise health inequalities.

**Supplementary Information:**

The online version contains supplementary material available at 10.1186/s12889-025-22248-8.

## Introduction

 The parental leave scheme in Sweden is considered one of the most generous and flexible in the world, where all registered residents have access to 480 days of paid parental leave per child until the child turns twelve years old or has completed grade five of compulsory education (Table 1 displays an overview of Sweden’s parental leave scheme) [1]. However, remuneration amounts are dependent on the individual’s income and participation in the labour market. Today, to obtain the most generous earnings-related parental leave benefit, a person must be employed for at least 240 consecutive days prior to the estimated delivery date and have a minimum annual income of 85 000 SEK (GBP 6330). Parents who meet this criterion receive almost 80% of their salary for 390 days with the remaining 90 days compensated at a minimum level (180 SEK/day; GBP 13·40/day) [1]. Many employees receive an additional ten percent supplement from their employers via collective agreement [2]. In contrast, parents who are outside the labour market or who have an income below the threshold for earnings-related parental leave benefits receive a basic pre-tax benefit of 7500 SEK (GBP 560) per month for 390 days and then the minimum level benefit thereafter [1].

**Table 1:** Overview of the paid parental leave in Sweden [1]

Parental leave (Swedish: *föraldraledighet*)	Parents in employment are entitled to job-protected leave of absence until the child reaches 18 months of age
Parental allowance (Swedish: *föraldrapenning*)	Parental allowance is paid for 480 days for a child. For 390 days, the compensation is based on the parent’s income (earnings-related benefit) and for the other 90 days, the compensation is a minimum-level benefit (180 SEK per day) The first 180 days taken out for the child must be days at earnings-related level. When you have taken out 180 days at the earnings-related level, you can start taking out days at the minimum-level as well Parents with joint custody are automatically assigned half of the parental leave benefit period (i.e., 240 days each), but days can be transferred to the other parent. From 2002, 60 days were reserved for each parent (often referred to as the ‘mother’ or ‘fathers’ quota). From 2016 until present, 90 days are reserved for each parent which cannot be transferred
Eligibility to parental allowance	All parents who are resident in Sweden are entitled to receive parental allowance if: • Taking care of a child • Not working, looking for employment or studying • The child lives in Sweden, in another country within the EU/EEA, or in Switzerland • The parent is insured in Sweden
Remuneration	The parental leave allowance is dependent on the parent’s income and length of employment prior to expected delivery date *Compensation based on income (390 days)* • If you have worked for at least 240 consecutive days prior to the expected birth and met an annual income threshold, then parents receive 77.6% of their salary for 390 days *(earnings-related benefits)* • If parents have not worked for at least 240 consecutive days before the expected birth, then they receive a basic flat-rate benefit for 390 days (*basic benefit*). From 2004, the basic benefit was set at 180 SEK per day, this was increased in 2013 to 225 SEK per day, and in 2016 it was further increased to 250 SEK per day where it currently stands *Minimum level benefit (Swedish: lägstanivå*) *(90 days)* From 2006, the minimum-level benefit is 180 SEK per day and has remained at this rate since
Flexibility	For children born before 1 January 2014, parents could withdraw parental allowance until the child turned 8 years of age or completed Grade 1 of primary education For children born from 1 January 2014, parents can withdraw parental allowance up to and including the day the child turns 12 years of age or until they have completed grade 5 of primary education. However, from the child’s fourth birthday only 96 days can be used
Responsibility	The Ministry of Social Affairs has the responsibility over the parental benefit, and the Ministry of Employment has the responsibility over the legislated right to leave from work
Funder	The Swedish Social Insurance Agency (Swedish: *Försäkringskassan*)
Formal childcare outside the home	In Sweden, children are entitled to attend preschool (Swedish: förskola) once they have turned one year old. As a result, the responsibility for childcare during the child’s first year falls on the parents. The flexibility of the parental leave policy allows parents to use their leave as best suited to their family needs

*Abbreviations*: *EEA* European Economic Area, *EU* European Union

A recent systematic review of the international literature concluded that parental leave generosity, in terms of remuneration and duration, is protective for mothers’ mental health in the postpartum period and beyond, including mothers with mental health conditions prior to pregnancy [3]. However, the review also suggested that work requirements (e.g., continuous employment for a fixed period of time prior to childbirth, meeting an annual income threshold) as a pre-requisite for more generous parental leave entitlements could be inadvertently responsible for increasing health inequalities in society, yet there is a lack of empirical evidence. Postpartum health inequalities can arise because certain groups, including those with ill-health [4] and migrants [5, 6], may also have weaker labour market attachment, and thus are less likely to benefit from the protective health effects of parental leave.

Although any health issue can potentially affect an individual’s capacity for employment, mental disorders and musculoskeletal conditions are the most common reasons for sickness absence in Sweden and elsewhere [7, 8]. In Sweden, among women of childbearing age, mental disorders account for approximately 60% of all work absenteeism cases, while the prevalence of absenteeism due to musculoskeletal conditions ranges from eight percent among women under 40 years of age to 13% in those aged 40–49 years [9]. Therefore, it is postulated that women who have suffered from these conditions may, in turn, be less likely to qualify for more generous parental leave entitlements due to weaker labour market attachment.

The aim of this study was to investigate the association between poor health in mothers prior to pregnancy and their eligibility for earnings-related parental leave benefits in Sweden in order to shed light on the extent to which the qualifying work requirement may act as a barrier for more generous parental leave benefits for mothers with pre-existing or chronic health conditions.

## Methods

This total population cohort study was performed as part of a larger project on the *Unintended consequences of the Swedish Parental Leave Policy (ParLeHealth): A health equity perspective*with a published peer-reviewed protocol [10].

### Data sources and study population

We used data from Swedish national registers that were linked using pseudonymised unique identification numbers. These registers included the Total Population Register, the Multi-Generation Register, and the Medical Birth Register, to construct our study population and identify first-time mothers; the National Patient Register, for information on hospitalisation and specialist outpatient care; the Swedish Social Insurance Agency (*Försäkringskassan*), for data on compensation level for parental leave remuneration benefits; the Longitudinal Integrated Database for Health Insurance and Labour Market Studies (LISA), for socioeconomic information, and the Geography Database for data on urbanicity.

**Fig. 1:**
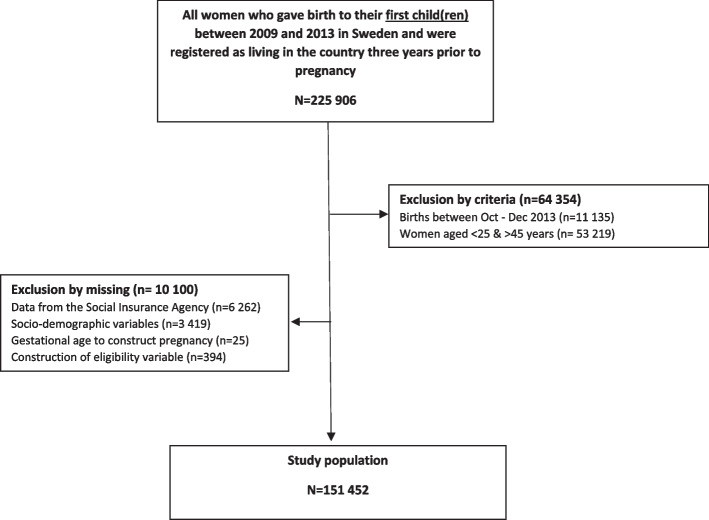
Study selection

The study population included all first-time mothers who gave birth in Sweden between 2009 and 2013, and who were resident in the country three years prior to pregnancy (*n* = 225 906). We restricted our population to mothers aged between 25 and 45 years to ensure adequate time in the labour market (*n* = 172 687), and excluded mothers whose child(ren) were born between October and December 2013 due to a reporting system change at the Swedish Social Insurance Agency (*n* = 11 135). We also excluded 10 100 mothers because of missing data. The final analytic sample consisted of 151 452 mothers (Fig. 1).

The study was approved by the Swedish Ethical Review Authority (2019–04913).

### Exposures

Using the 10th edition of the International Classification of Diseases (ICD) codes, our exposures included assessment of four health categories: any health condition (all ICD chapters excluding external causes for morbidity and mortality: V01-Y98); mental disorders (F00-F99); musculoskeletal conditions (M00-M99); and other health conditions (excluding mental disorders and musculoskeletal conditions). These health categories were assessed across two levels of healthcare use representing different levels of severity: hospitalisation; and specialist outpatient care (hereinafter outpatient care) as well as a combined category: hospitalisation or specialist outpatient care (hereinafter any healthcare treatment). A complete list of all ICD-10 codes and their definition are in Table A1 (Appendix 1). Exposures were dichotomous (e.g., any health condition versus none) and measured in the year (365 days) prior to pregnancy. In separate analyses, a proxy measure was used to assess the presence of chronic conditions by examining healthcare utilisation for two consecutive years before pregnancy. Health conditions were identified by primary diagnosis to focus on the principal health issue, thereby avoiding confounding with secondary conditions. Using primary diagnoses ensures that the population is selected based on consistent criteria, enhancing comparability across studies and improving replicability.

### Outcome

We assessed eligibility for earnings-related parental leave benefits using the compensation level variable for parental leave remuneration determined by the Swedish Social Insurance Agency when the mother applied for parental level benefits for the first time after childbirth. Eligibility was categorised into two groups: recipients of earnings-related benefit payments (generous) and recipients of basic flat-rate payments (basic), i.e., those who were not eligible for earnings-related benefits.

### Socio-demographic variables

We obtained birth year of the child and mother’s age at the time of birth, measured continuously in completed years, from the Medical Birth Register. Using data from the Total Population Register, we classified mothers based on their *region *of birth into three groups: 1) from Sweden, 2) from regions primarily composed of Organisation for Economic Cooperation and Development (OECD) member countries including the Nordic region (excluding Sweden), Europe, North America and Oceania, and 3) from non-OECD regions primarily comprising of mothers from Africa, Middle East, Asia, South America, or stateless/unspecified regions [11]. Our socio-economic measure was highest level of education attained by the mother at approximately two years prior to pregnancy. Mother’s educational level was categorised as *low* (compulsory schooling: up to nine years of education), *medium* (upper-secondary schooling: up to 12 years of education), and *high* (any tertiary education)*.*

### Statistical analysis

All analyses were performed using Stata v17 [12]. Logistic regression models were used to calculate odd ratios (OR) and 95% Confidence Intervals (95% CI). Health prior to pregnancy was assessed in separate analyses, and were specific for each health complaint, whereby those without that specific health issue were included in the comparison group. All analyses adjusted for birth year of the child to account for cohort effects, and mother’s age at birth (Model 1). We further adjusted for mother’s education (Model 2), in order to ascertain if potential differences in the main association were explained by education. Furthermore, we performed stratified subgroup analyses by mother’s region of birth to ascertain if potential differences in the main effect were driven by birthplace. In addition to odds ratios, we also estimated average marginal effects (AME) for these analyses. In Sweden, the health care system is universal, primarily government funded, decentralised, and managed by regions, local authorities or municipalities. Therefore, we do not expect area of residence (*urbanicity*) to substantially influence access to healthcare. However, we conducted sensitivity analyses to test this assumption (see Appendix 2 for description and operationalisation of this variable). As hospitalisation and outpatient care variables were not mutually exclusive, and may have overlapped, we also performed sensitivity analyses excluding mothers who were hospitalised i.e., those with more severe illness, from the outpatient care variable.

## Results

The mean age of mothers at the time of birth was 30·5 years (SD 3·9) (Table 2). The majority (94·2%) were eligible for earnings-related parental leave benefits, born in Sweden (88·2%) and highly educated (62·6%). Mothers with low level of education attainment (74·4%) had lower eligibility for earnings-related benefits. The proportion of mothers eligible for earnings-related benefits was highest among Swedish-born mothers (95%), and lowest among mothers from non-OECD regions (80%). The prevalence of any health conditions was lowest among Swedish-born mothers (41%), and ranged from 43% for mothers from OECD regions to 47% for mothers from non-OECD regions.

**Table 2:** Characteristics of the study population (*N* = 151 452)

			**Eligible for earning’s-related parental leave benefits**		
	**N (SD)**	**Range/%**	**N**	**%**	***p*-value**
**Mother’s age at birth (years)**	30·5 (3·94)	25–45			
**Birth year of the child**					
2009	31 834	21·02	30 036	94·35	
2010	32 909	21·73	30 961	94·08	
2011	31 581	20·85	29 715	94·09	
2012	31 903	21·06	30 079	94·28	
2013	23 225	15·33	21 854	94·10	*p* = 0·45
**Educational level**					
Low	6 742	4·45	5 015	74·38	
Medium	49 984	33·00	46 444	92·92	
High	94 726	62·55	91 186	96·26	*p* < 0·001
**Region of birth**					
Sweden	133 527	88·16	127 494	95·48	
Migrant from OECD	8 451	5·58	7 592	89·84	
Migrant from Non-OECD	9 474	6·26	7 559	79·79	*p* < 0·001
**Eligible for earnings related benefits**					
Eligible to earnings-related benefits	142 645	94·18			
Not eligible to earnings-related benefits	8 807	5·83			
***Health in year prior to pregnancy***					
**Any health condition**					
Any healthcare treatment	63 398	41·86	59 343	93·60	
No healthcare treatment	88 054	58·14	83 302	94·60	*p* < 0·001
Specialist outpatient care	62 216	41·08	58 248	93·62	
No specialist outpatient care	89 236	58·92	84 397	94·58	*p* < 0·001
Hospitalisation	8 095	5·34	7 384	91·22	
No hospitalisation	143 357	94·6	135 261	94·35	*p* < 0·001
**Mental disorder**					
Any healthcare treatment	3 828	2·53	3 029	79·13	
No healthcare treatment	147 62	97·47	139 616	94·58	*p* < 0·001
Specialist outpatient care	3 687	2·43	2 938	79·69	
No specialist outpatient care	147 765	97·57	139 707	94·55	*p* < 0·001
Hospitalisation	457	0·30	297	64·99	
No hospitalisation	150 995	99·70	142 348	94·27	*p* < 0·001
**Musculoskeletal condition**					
Any healthcare treatment	4 888	3·23	4 633	94·78	
No healthcare treatment	146 564	96·77	138 012	94·17	*p* = 0·07
Specialist outpatient care	4 814	3·18	4 563	94·79	
No specialist outpatient care	146 638	96·82	138 082	94·17	*p* = 0·07
Hospitalisation	272	0·18	256	94·12	
No hospitalisation	151 180	99·82	142 389	94·19	*p* = 0·96
**Other health conditions**					
Any healthcare treatment	59 891	39·54	56 195	93·83	
No healthcare treatment	91 561	60·46	86 450	94·42	*p* < 0·001
Specialist outpatient care	58 658	38·73	55 059	93·86	
No specialist outpatient care	92 794	61·27	87 586	94·39	*p* < 0·001
Hospitalisation	7 521	4·97	6 935	92·21	
No hospitalisation	143 931	95·03	135 710	94·29	*p* < 0·001
***Health two years prior to pregnancy (chronic health***					
**Any health condition**					
Any healthcare treatment	33 427	22·07	30 886	92·40	
No healthcare treatment	118 025	77·93	111 759	94·69	*p* < 0·001
Specialist outpatient care	32 527	21·48	30 086	92·5	
No specialist outpatient care	118 925	78·52	112 559	94·65	*p* < 0·001
Hospitalisation	1 080	0·71	897	83·06	
No hospitalisation	150 372	99·29	141 748	94·26	*p* < 0·001
**Mental disorder**					
Any healthcare treatment	1 929	1·27	1 426	73·92	
No healthcare treatment	149 523	98·73	141 219	94·45	*p* < 0·001
Specialist outpatient care	1 843	1·22	1 372	74·44	
No specialist outpatient care	149 609	98·78	141 273	94·43	*p* < 0·001
Hospitalisation	117	0·08	57	48·72	
No hospitalisation	151 335	99·92	142 588	94·22	*p* < 0·001
**Musculoskeletal condition**					
Any healthcare treatment	1 550	1·02	1 456	93·94	
No healthcare treatment	149 902	98·98	141 189	94·19	*p* = 0·67
Specialist outpatient care	1 508	1·00	1 417	93·97	
No specialist outpatient care	149 944	99·00	141 228	94·19	*p* = 0·71
Hospitalisation	29	0·02	26	89·66	
No hospitalisation	151 423	99·98	142 619	94·19	*p* = 0·30
**Other health conditions**					
Any healthcare treatment	29 780	19·66	27 693	92·99	
No healthcare treatment	121 672	80·34	114 952	94·48	p < 0·001
Specialist outpatient care	28 883	19·07	26 890	93·10	
No specialist outpatient care	122 569	80·93	115 755	94·44	*p* < 0·001
Hospitalisation	863	0·57	750	86·91	
No hospitalisation	150 589	99·43	141 895	94·23	*p* < 0·001

Household income and educational level measured approximately two years prior to pregnancy

**Fig. 2:**
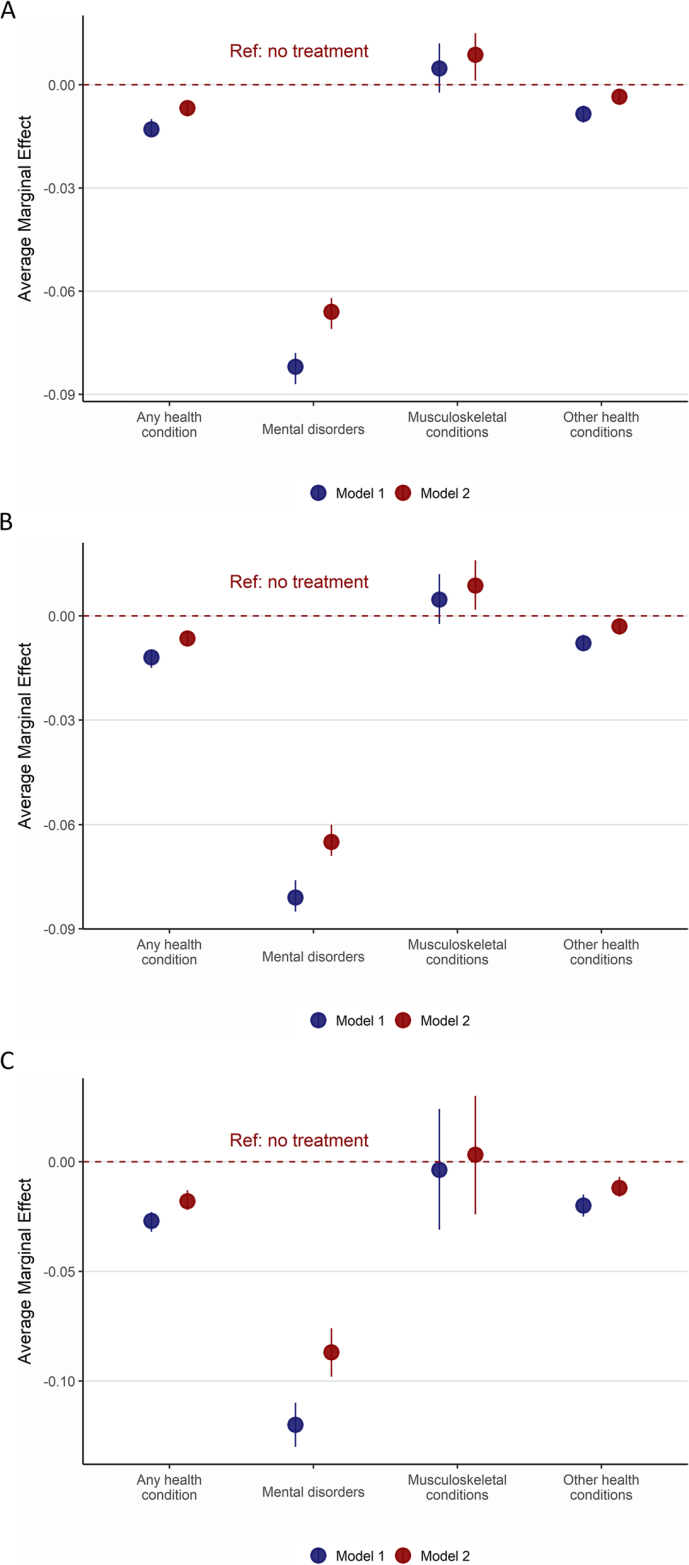
The average marginal effects with 95% confidence intervals of logistic regression exploring the association between any health condition; mental disorders; musculoskeletal conditions; and other health conditions; and eligibility for earnings-related parental leave benefits in the year prior to pregnancy (*N* = 151 452). **A**: Any healthcare treatment, **B**: Outpatient care, and **C**: Hospitalisation

Mothers receiving any healthcare treatment for any health condition in the year before pregnancy had lower odds of being eligible for earnings-related benefits (OR 0·79, 95%CI 0·76–0·83; Model 1) compared to mothers with no healthcare treatment (Table 3). The estimate was similar for mothers receiving outpatient care (OR 0·80, 95%CI 0·77–0·84; Model 1). However, mothers hospitalised for any health condition had even lower odds of being eligible for earnings-related benefits compared to mothers who were not hospitalised for any health condition (OR 0·60, 95% CI 0·56–0·65; Model 1). Mothers who received any healthcare treatment for mental disorders in the year preceding pregnancy had significantly decreased odds of being eligible for earnings-related benefits compared with mothers who did not receive treatment for mental disorders (OR 0·22, 95%CI 0·20–0·23; Model 1). Mothers admitted to hospital for mental disorders had even lower odds for eligibility for earnings-related benefits compared to mothers who were not hospitalised for mental disorders (OR 0·12, 95% CI 0·10–0·14; Model 1). Moreover, mothers receiving outpatient care or hospitalised for *other* health conditions also demonstrated significantly lower odds of being eligible for earnings-related benefits compared with healthy mothers (OR 0·87, 95%CI 0·83–0·90 for outpatient care (Model 1); OR 0·70, 95%CI 0·64–0·76 for hospitalisation (Model 1)). In contrast, there was no evidence of an association between receiving any healthcare treatment for musculoskeletal conditions in the year prior to pregnancy and being eligible for earnings-related parental leave benefits (OR 1·09, 95%CI 0·96–1·24; Model 1) (Table 3). Figure 2 and Table 3(Appendix 3) displays the average marginal effects in the association between health prior to pregnancy and eligibility for earnings-related parental leave benefits to facilitate comparison with odd ratio estimates.

**Table 3:** The association between any health condition; mental disorders; musculoskeletal conditions; and other health conditions; and eligibility for earnings-related parental leave benefits (*N* = 151 452)

Health in the year prior to pregnancy							Health in the two consecutive years prior to pregnancy (chronic health)					
	**Any healthcare treatment**		**Specialist outpatient Care**		**Hospitalisation**		**Any healthcare treatment**		**Specialist outpatient Care**		**Hospitalisation**	
	**Model 1** **OR (95% CI)**	**Model 2** **OR (95% CI)**	**Model 1** **OR (95% CI)**	**Model 2** **OR (95% CI)**	**Model 1** **OR (95% CI)**	**Model 2** **OR (95% CI)**	**Model 1** **OR (95% CI)**	**Model 2** **OR (95% CI)**	**Model 1** **OR (95% CI)**	**Model 2** **OR (95% CI)**	**Model 1** **OR (95% CI)**	**Model 2** **OR (95% CI)**
No health condition	1	1	1	1	1	1	1	1	1	1	1	1
Any health condition	0·79 (0·76–0·83) ***	0·88 (0·84–0·92) ***	0·80 (0·77–0·84) ***	0·88 (0·85–0·92) ***	0·60 (0·56–0·65) ***	0·71 (0·66–0·77) ***	0·64 (0·62–0·68) ***	0·74 (0·71–0·78) ***	0·66 (0·63–0·69) ***	0·76 (0·72–0·80) ***	0·29 (0·25–0·34) ***	0·41 (0·35–0·49) ***
No mental disorders	1	1	1	1	1	1	1	1	1	1	1	1
Any mental disorders	0·22 (0·20–0·23) ***	0·28 (0·26–0·31) ***	0·22 (0·21–0·24) ***	0·29 (0·26–0·32) ***	0·12 (0·10–0·14) ***	0·19 (0·15–0·23) ***	0·16 (0·15–0·18) ***	0·22 (0·20–0·25) ***	0·17 (0·15–0·19) ***	0·22 (0·20–0·25) ***	0·06 (0·04–0·09) ***	0·11 (0·08–0·17) ***
No musculoskeletal condition	1	1	1	1	1	1	1	1	1	1	§	§
Any musculoskeletal condition	1·09 (0·96–1·24)	1·18 (1·03–1·34) *	1·09 (0·96–1·24)	1·18 (1·04–1·35) *	0·93 (0·56–1·55)	1·06 (0·63–1·78)	0·91 (0·74–1·13)	0·99 (0·80–1·23)	0·92 (0·74–1·14)	0·99 (0·80–1·23)		
No other health condition	1	1	1	1	1	1	1	1	1	1	1	1
Any other health condition	0·86 (0·82–0–89) ***	0·94 (0·89–0·98) **	0·87 (0·83–0·90) ***	0·94 (0·90–0·99) *	0·70 (0·64–0·76) ***	0·80 (0·73–0·88) ***	0·74 (0·70–0·77) ***	0·83 (0·79–0·88) ***	0·75 (0·71 −0·79) ***	0·85 (0·81–0·90) ***	0·40 (0·32–0·48) ***	0·53 (0·43–0·65) ***

Model 1: Adjusted for child’s year of birth, mother’s age

Model 2: Adjusted for child’s year of birth, mother’s age, education

*OR* Odds ratio, *95%CI* 95% confidence intervals

^§^Not enough units in cell to perform analysis for hospitalization

^*^p < 0·05

^**^p < 0·01

^***^p < 0·001

The inclusion of education in the models largely did not influence the pattern of associations, with the exception of musculoskeletal conditions. Mothers who received outpatient care for such conditions were more likely to be eligible for earnings-related benefits (OR 1.18, 95%CI 1.03–1.34; Model 2) (Table 3).

Moreover, the odds of being eligible for earnings-related parental leave benefits were further reduced among mothers receiving healthcare treatment for any health condition, mental disorders, or other health conditions in both of the two consecutive years prior to pregnancy, compared to mothers who did not receive any healthcare treatment during those years. This was particularly apparent among mothers who received healthcare treatment for mental disorders (OR 0·16, 95%CI 0·15–0·18; Model 1). Odds ratio estimates were reduced further among those who were hospitalised for mental disorders (OR 0·06 95%CI 0·04–0·09; Model 1). When analyses were further adjusted by education, similar patterns remained (Table 3).

### Sub-analyses

Receiving healthcare treatment for *any* and *other* health conditions differed by region of origin (Table 4). The proportion of mothers receiving outpatient care and being admitted to hospital was lowest among Swedish born mothers (41·38% and 38·19%), followed by mothers from OECD regions (43·32% and 41·52%), and mothers from non-OECD regions (47·26% and 45·26%), for *any* and *other* health conditions, respectively. In contrast, the proportion of mothers receiving healthcare treatment for mental disorders and musculoskeletal conditions was similar across region of origin groups, and ranged from 2·1–2·6% for mental disorders and 2·91–3·25% for musculoskeletal conditions.

**Table 4:** The association between any health condition; mental disorders; musculoskeletal conditions; and other health conditions in the year prior to pregnancy; and eligibility for earnings-related parental leave benefits by region of origin (*n* = 151·452)

	**Born in Sweden (*n* = 133 527)**				**Born in OECD regions (*n* = 8 451)**				**Born in non-OECD regions (*n* = 9 474)**			
	**N**	**%**	**Model 1** **OR (95% CI)**	**Model 2** **OR (95% CI)**	**N**	**%**	**Model 1** **OR (95% CI)**	**Model 2** **OR (95% CI)**	**N**	**%**	**Model 1** **OR (95% CI)**	**Model 2** **OR (95% CI)**
**Any health condition**												
No healthcare treatment	78 267	58·62	1	1	4 790	56·68	1	1	4 997	52·74	1	1
Any healthcare treatment	55 260	41·38	0·80 (0·76–0·84) ***	0.88 (0.84–0.93) ***	3 661	43·32	0·87 (0·75–1·00) *	0.94 (0.82–1.09)	4 477	47·26	0·91 (0·82–1·00)	0.96 (0.87–1.07)
No outpatient care	79 319	59·40	1	1	4 837	57·24	1	1	5 080	53·62	1	1
Outpatient care	54 208	40·60	0·80 (0·76–0·85) ***	0.89 (0.84–0.93) ***	3 614	42·76	0·87 (0·75–1·00)	0.95 (0.82–1.10)	4 394	46·38	0 ·92 (0·83–1·02)	0.97 (0.88–1.08)
No hospitalisation	126 581	94·80	1	1	7 967	94·27	1	1	8 809	92·98	1	1
Hospitalisation	6 946	5·20	0·59 (0·54–0·65) ***	0.69 (0.63–0.77) ***	484	5·73	0·68 (0·52–0·90) **	0.77 (0.58–1.01)	665	7·02	0·78 (0·65–0·94) *	0.86 (0.71–1.04)
**Mental disorders**												
No healthcare treatment	130 123	97·45	1	1	8 273	97·89	1	1	9 228	97·40	1	1
Any healthcare treatment	3 404	2·55	0·18 (0·16–0·19) ***	0.23 (0.21–0.25) ***	178	2·11	0·37 (0·26–0·53) ***	0.45 (0.31–0.65) ***	246	2·60	0·39 (0·30–0·51) ***	0.43 (0.33–0.57) ***
No outpatient care	130 239	97·54	1	1	8 283	98·01	1	1	9 243	97·56	1	1
Outpatient care	3 288	2·46	0·18 (0·17–0·20) ***	0.24 (0.21–0.26) ***	168	1·99	0·42 (0·29–0·62) ***	0.51 (0.34–0.76) **	231	2·44	0·42 (0·32–0·55) ***	0.45 (0.34–0.60) ***
No hospitalisation	133 134	99·71	1	1	8 427	99·72	1	1	9 434	99·58	1	1
Hospitalisation	393	0·29	0·10 (0·08–0·12) ***	0.16 (0.13–0.20) ***	24	0·28	0·20 (0·08–0·45) ***	0.25 (0.11–0.60) **	40	0·42	0·25 (0·13–0·47) ***	0.35 (0.18–0.67) **
**Musculoskeletal conditions**												
No healthcare treatment	129 191	96·75	1	1	8 205	97·09	1	1	9 168	96·77	1	1
Any healthcare treatment	4 336	3·25	1·00 (0·86–1·16)	1.08 (0.93–1.26)	246	2·91	1·01 (0·67–1·55)	1.18 (0.76–1.83)	306	3·23	1·58 (1·13–2·20) **	1.59 (1.14–2.23) **
No outpatient care	129 256	96·80	1	1	8 207	97·11	1	1	9 175	96·84	1	1
Outpatient care	4 271	3·20	1·00 (0·86–1·16)	1.08 (0.93–1.25)	244	2·89	1·01 (0·66–1·54)	1.17 (0.76–1.82)	299	3·16	1·63 (1·16–2·28) **	1.65 (1.17–2.33) **
No hospitalisation	133 281	99·82	1	1	8 440	99·87	1	1	9 459	99·84	1	1
Hospitalisation	246	0·18	0·93 (0·51–1·71)	1.09 (0.89–2.01)	11	0·13	1.08 (0.13–8.49)	1.32 (0.16–10.73)	15	0·16	0.70 (0.22–2.19)	0.67 (0.21–2.15)
**Other health conditions**												
No healthcare treatment	81 433	60·99	1	1	4 942	58·48	1	1	5 186	54·74	1	1
Any healthcare treatment	52 094	39·01	0·89 (0·85–0·94) ***	0.97 (0.92–1.03)	3 509	41·52	0·88 (0·76–1·02)	0.96 (0.83–1.11)	4 288	45·26	0·91 (0·82–1·01)	0.97 (0.87–1.07)
No outpatient care	82 528	61·81	1	1	4 992	59·07	1	1	5 274	55·67	1	1
Outpatient care	50 999	38·19	0·90 (0–85-0·95) ***	0.98 (0.93–1.03)	3 459	40·93	0·89 (0·77–1·03)	0.97 (0.84–1.12)	4 200	44·33	0·93 (0·84–1·03)	0.98 (0.88–1.09)
No hospitalisation	127 093	95·18	1	1	7 990	94·55	1	1	8 848	93·39	1	1
Hospitalisation	6 434	4·82	0·72 (0·64–0·80) ***	0.82 (0.73–0.92) ***	461	5·45	0·71 (0·54–0·94) *	0.80 (0.60–1.07)	626	6·61	0·82 (0·68–1·00) *	0.88 (0.72–1.08)

Reference category is no health condition, mental disorder· musculoskeletal condition or other health condition

Model 1: Adjusted for child’s year of birth mother’s age

Model 2: Adjusted for child’s year of birth mother’s age, education

*OR* Odds ratio, *95%CI* 95% Confidence intervals

^*^p < 0·05

^**^p < 0·01

^***^p < 0·001

Sub-analyses by origin (Table 4, Tables A4 and A5, Appendices 4 and 5) further showed that associations were typically strongest among Swedish-born mothers. Among mothers receiving healthcare treatment for any health condition, the odds of being eligible for earnings-related benefits were 0·80 (95%CI 0·76–0·84; Model 1) among Swedish-born mothers, 0·87 (95%CI 0·75–1·00; *p* < 0.05; Model 1) among mothers from OECD regions, and 0·91 (95%CI 0·82–1·00; *p* = 0.059; Model 1) among mothers from non-OECD regions, relative to their counterparts who did not receive healthcare treatment.

While mothers receiving healthcare treatment for mental disorders from all groups had significantly lower odds of being eligible for earnings-related benefits compared to their healthy counterparts, this association was most pronounced among Swedish-born mothers (OR 0·18, 95%CI 0·16–0·19). The odds of being eligible for earnings-related benefits was similar among foreign-born mothers (OR 0·37, 95%CI 0·26–0·53 for those from OECD regions (Model 1) versus OR 0·39, 95%CI 0·30–0·51 for those from non-OECD regions (Model 1)).

Similar to the main analysis, there was no evidence of an association between receiving treatment for musculoskeletal conditions in the year prior to pregnancy and being eligible for earnings-related benefits for Swedish-born mothers and mothers from OECD regions. However, the odds of being eligible for earnings-related benefits *increased* among mothers receiving outpatient care for musculoskeletal conditions who were from non-OECD regions (OR 1·63 95%CI 1·16–2·28; Model 1) compared with mothers from non-OECD regions who did not receive care. When education was included into the model, both Swedish-born mothers and mothers from OECD regions also had increased odds of being eligible for earnings-related benefits if they received treatment for musculoskeletal conditions.

### Sensitivity analyses

We also examined the potential role of urbanicity in the association between health in the year before pregnancy and chronic health and eligibility for earnings-related parental leave benefits, and found no change in odd ratios estimates when urbanicity was included in our models (results not shown). Exclusion of mothers who were hospitalised (i.e., those with more severe illnesses) from analyses of outpatient care demonstrated estimates that were very similar to those observed in the main analyses (Table A6, Appendix 6).

## Discussion

Our findings indicated that mothers who were hospitalised and/or received outpatient care before pregnancy, particularly for mental disorders, were less likely to be eligible for earnings-related parental leave benefits compared to mothers who did not receive healthcare treatment. Moreover, the likelihood of being eligible for more generous benefits was decreased further for mothers receiving healthcare treatment in the two consecutive years prior to pregnancy (indicative of chronic problems). Overall, no difference was observed between mothers with and without musculoskeletal conditions; however, mothers from non-OECD regions receiving outpatient care for musculoskeletal conditions were more likely to be eligible for earnings-related benefits.

In our study, mental health, rather than physical health, prior to pregnancy had a greater impact on eligibility for earnings-related parental leave benefits, suggesting that mechanisms explaining the relationship between health and labour market attachment may vary based on specific health outcomes. For example, some musculoskeletal conditions (e.g., back pain and neck strain) may temporarily prevent individuals from working with minimal impact on job security, whereas individuals with mental disorders may face greater challenges in the workplace, especially without supportive accommodations such as flexible hours and health-related leave [13]. Furthermore, individuals with mental disorders may also encounter greater difficulties in securing employment [14, 15].

Our findings are also in line with the accumulation of risk model [16], as we observed that mothers with chronic health conditions, particularly mental disorders, had even lower odds of being eligible for earnings-related parental leave benefits. This suggests that eligibility for more generous benefits may reinforce inequalities that existed before pregnancy.

In Sweden, healthcare access is universal for all residents; however, utilisation differs between native-born and foreign-born individuals, a trend observed in many high-income countries [17]. Evidence suggests that migrants typically use less psychiatric healthcare than Swedish-born individuals, particularly during their first years in the country [18]. However, utilisation among migrants increases with time, and those who have been in Sweden for more than a decade generally exhibit higher psychiatric care use. Additionally, psychiatric health care use among migrants varies depending on their region of origin [18].

We observed that mothers from non-OECD regions receiving outpatient care for musculoskeletal conditions remained in the labour market while undergoing treatment, leading to higher odds of being eligible for earnings-related benefits. This observation aligns with prior research [19, 20] where migrants tend to engage in more physically demanding jobs compared to native-born workers, and continue employment despite physical ailments. A similar pattern, albeit less pronounced, was observed for mothers from other origins when education was taken into consideration. It appears that highly educated mothers in all groups tend to remain in the labour market to secure earnings-related benefits, possibly being aware of the consequences of not being employed during this life stage. Furthermore, we observed an increase in the odds of eligibility only among mothers who received healthcare treatment in the year before pregnancy, but not among mothers with chronic musculoskeletal conditions. This observation may suggest that musculoskeletal conditions in the year before pregnancy are milder, allowing women to continue employment.

Furthermore, our findings showed that immigrant mothers, particularly those from non-OECD regions, were less likely to qualify for earnings-related benefits than Swedish-born mothers. Nonetheless, the associations between pre-pregnancy healthcare treatment and eligibility for earnings-related benefits were more pronounced among Swedish-born mothers, even though all mothers receiving healthcare treatment, regardless of their origin, had lower odds of eligibility for more generous benefits. This may indicate that immigrant mothers are more likely to remain active in the labour market even when they are unwell, a trend observed in other settings [21, 22].

The Swedish parental leave scheme is renowned for its generosity; however, as this study shows, the relatively strong work requirement for earnings-related parental leave benefits might be an unintentional source of economic inequalities, and further health inequalities. Thus, the health inequalities observed in this study could be larger in contexts with more stringent work requirements. For example, in some European countries, self-employed individuals do not receive the same entitlements as employed individuals; in the United Kingdom, self-employed individuals are not eligible to paid parental leave [23], while in Italy, self-employed parents are not entitled to the same length of paid leave as those employed (3 versus 6 months) [24]. Furthermore, even though the proportion of mothers who were not eligible for earnings-related benefits was relatively low (around 6%) in our study, the rising prevalence of mental disorders among young adults [25] and the increasing precariousness of the labour market [26], where temporary contracts continue to grow, suggest that inequalities in access to the most generous benefits can increase if strict conditions remain [27].

The main strength of the study is that we used data from Swedish total population and health registers, which ensures representativeness. Furthermore, by relying on medical records, we were able to avoid recall bias. The study does have some limitations. Firstly, we only included mothers with complete data. However, the proportion of missing data was low (only 6·3%). Secondly, although our health measures capture more severe health outcomes, such as hospitalisations, individuals in Sweden can self-refer to specialised outpatient care for mental disorders. Of adults receiving mental health care, 49% are treated in primary care, 32% in secondary care, and 18.5% are jointly treated in both primary and secondary care [28]. Accordingly, our measure was possibly able to capture milder mental health complaints in outpatient care. This may explain the observed association between health and eligibility for generous parental leave benefits even when there is likely some health-based selection into motherhood. Thirdly, we were not able to use a more detailed categorisation of birthplace in order to maintain enough statistical power; however, our categorisation matches those used in previous studies, ensuring comparability [11, 29]. Fourthly, our results were robust across different likelihood estimates (ORs and AMEs). Finally, despite using longitudinal data and being able to determine the temporal order of the events, we cannot rule out the possibility that a weaker attachment to the labour market is responsible for a mother’s poor health rather than the other way around.

To the best of our knowledge, this study is the first to evaluate whether there is health selection into earnings-related parental leave benefits, both in Sweden and worldwide. Consequently, comparisons with previous studies are not possible. The results of our study can inform policy makers to design much-needed equitable parental leave systems [30]. Our findings support the need to embrace a *Health in all Policies*approach, whereby health implications are considered when designing social interventions [31]. This framework facilitates an examination of structural factors that lead to health inequalities, and its adoption can enable an improved public health and health equity.

Generous parental leave has been shown to be protective for mother’s mental health including those with pre-existing mental health conditions [3]. Consequently, the fact that mothers with mental disorders are less likely to be eligible for more generous parental leave remunerations due to weaker labour market attachment [4] implies that the design of the parental leave policy, through its eligibility criteria, may contribute to increasing social and health inequalities in society. In Sweden, there is no formal childcare available for children under twelve months of age, which may place financial strain on parents during this period, potentially increasing health burdens [32, 33], and compromising gender equity, as fathers typically hold better positions in the labour market. Relaxing some of the stringent work requirements for eligibility for earnings-related parental leave benefits may contribute to reducing health inequities in society while promoting gender equity across social groups. In the Swedish context, this could be achieved by extending the work eligibility period, similar to the Norwegian model, where eligibility is based on being employed for six out of the last ten months before the birth and earnt above a minimum income level [34].

Moreover, mothers with access to childcare may return to work sooner than those with more generous benefits, as the basic benefit is insufficient as a living wage. This may result in less time for post-pregnancy recovery, reduced opportunities for bonding, and potentially suboptimal breastfeeding practices [35]. Furthermore, mothers with weak labour market attachment or low earnings may delay starting a family until they are older and established in the labour market, increasing the chances of experiencing age-related problems during pregnancy (including but not limited to maternal complications, such as pre-eclampsia, gestational diabetes and caesarean section) [36].

## Conclusion

This is the first study to examine associations between mother’s health prior to pregnancy and eligibility for earnings-related parental leave benefits. Mothers who received specialist outpatient care or were hospitalised before pregnancy, particularly for mental disorders, were less likely to be eligible for earnings-related parental leave benefits, thereby highlighting inequalities in accessing the most generous parental leave benefits.

## Supplementary Information

Supplementary Material 1.

## Data Availability

The datasets generated and/or analysed during the current study are not publicly available due to ethical approval but are available from the corresponding author on reasonable request.

## Abbreviations

95%CI: 95% Confidence Intervals
EEA: European Economic Area
EU: European Union
ICD: International Classification of Diseases
OECD: Organisation for Economic Cooperation and Development
OR: Odds ratio
