# Supplementary material for: Health before pregnancy and eligibility for parental leave benefits: a Swedish total population cohort study
Source: BMC Public Health. 2025 Mar 18;25:1045. doi: 10.1186/s12889-025-22248-8 (PMC11917127; doi:10.1186/s12889-025-22248-8)
Supplement: Supplementary file 1 — Supplementary Material 1. [file 12889_2025_22248_MOESM1_ESM.docx]

**Health before pregnancy and eligibility for parental leave benefits: a Swedish total population cohort study.**

Amy Heshmati* MSc,^1, 2, 3^ Andrea Dunlavy PhD,^1,2^ Eleonora Mussino PhD,^4^ Sara Fritzell PhD,^3^ and Sol P. Juárez PhD^1, 2^

1. Centre for Health Equity Studies (CHESS), Stockholm University/Karolinska Institutet, Stockholm, Sweden
2. Department of Public Health Sciences, Stockholm University, Stockholm, Sweden
3. Department of Global Public Health, Karolinska Institutet, Stockholm, Sweden
4. Stockholm University Demography Unit (SUDA), Stockholm, Sweden

*Corresponding author:

Amy Heshmati

Department of Public Health Sciences,

Stockholm University,

Albanovägen 12,

10 691 Stockholm,

Sweden.

Phone: +46 767 109 471

Email: [amy.heshmati@su.se](mailto:amy.heshmati@su.se)

**Contents**

Appendix 1. 10^th^ Edition of the International Classification of Disease Codes……………….…….[2](#Appendix1)

Appendix 2. Urbanicity description…………...…………………………………………………….[4](#Appendix2)

Appendix 3. Average Marginal Effects: health and eligibility for earnings-related benefits……….[5](#Appendix3)

Appendix 4. Chronic health and eligibility for earnings-related benefits by region of origin……....[6](#Appendix4)

Appendix 5. Average Marginal Effects: health and eligibility for earnings-related benefits
by region of origin……………………………………………………………………. [8](#Appendix5)

Appendix 6. Sensitivity analysis: outpatient care and eligibility for earnings-related benefits……[10](#Appendix6)

**Appendix 1**

| **Table A1. The 10^th^ edition of the International Classification of Disease Codes** | | |
| --- | --- | --- |
| **Chapter** | **Codes** | **Definition** |
| I | A00-B99 | Certain infectious and parasitic diseases |
| II | C00-D48 | Neoplasms |
| III | D50-D89 | Diseases of the blood and blood-forming organs and certain disorders involving the immune mechanism |
| IV | E00-E90 | Endocrine, nutritional and metabolic diseases |
| V | F00-F99 | Mental and behavioural disorders |
|  | F00-F09 | Organic, including symptomatic, mental disorders |
|  | F10-F19 | Mental and behavioural disorders due to psychoactive substance use |
|  | F20-F29 | Schizophrenia, schizotypal and delusional disorders |
|  | F30-F39 | Mood [affective] disorders |
|  | F40-F48 | Neurotic, stress-related and somatoform disorders |
|  | F50-F59 | Behavioural syndromes associated with physiological disturbances and physical factors |
|  | F60-F69 | Disorders of adult personality and behaviour |
|  | F70-F79 | Mental retardation |
|  | F80-F89 | Disorders of psychological development |
|  | F90-F98 | Behavioural and emotional disorders with onset usually occurring in childhood and adolescence |
|  | F99-F99 | Unspecified mental disorder |
| VI | G00-G99 | Diseases of the nervous system |
| VII | H00-H59 | Diseases of the eye and adnexa |
| VIII | H60-H95 | Diseases of the ear and mastoid process |
| IX | I00-I99 | Diseases of the circulatory system |
| X | J00-J99 | Diseases of the respiratory system |
| XI | K00-K93 | Diseases of the digestive system |
| XII | L00-L99 | Diseases of the skin and subcutaneous tissue |
| XIII | M00-M99 | Diseases of the musculoskeletal system and connective tissue |
|  | M00-M25 | Arthropathies |
|  | M30-M36 | Systemic connective tissue disorders |
|  | M40-M54 | Dorsopathies |
|  | M60-M79 | Soft tissue disorders |
|  | M80-M94 | Osteopathies and chondropathies |
|  | M95-M99 | Other disorders of the musculoskeletal system and connective tissue |
| XIV | N00-N99 | Diseases of the genitourinary system |
| XV | O00-O99 | Pregnancy, childbirth and the puerperium |
| (XVI | P00-P96 | Certain conditions originating in the perinatal period) |
| XVII | Q00-Q99 | Congenital malformations, deformations and chromosomal abnormalities |
| XVIII | R00-R99 | Symptoms, signs and abnormal clinical and laboratory findings, not elsewhere classified |
| XIX | S00-T98 | Injury, poisoning and certain other consequences of external causes |
| XX | V01-Y98 | External causes of morbidity and mortality |
| XXI | Z00-Z99 | Factors influencing health status and contact with health services |
| XXII | U00-U85 | Codes for special purposes |

Reference:

ICD-10 Version: 2019. Accessed September 16, 2024: <https://icd.who.int/browse10/2019/en>

**Appendix 2**

**Urbanicity description**

Urbanicity was based on the Demographic Statistical Area (DeSo) measure developed by Statistics Sweden. DeSo refers to the demographic statistics areas established by the government, each with between 700 and 2700 inhabitants. These areas are grouped into three categories: A includes areas primarily outside major population concentrations or urban areas (*rural*, n=15,635); B refers to areas mostly in a population concentration, but not areas centrally located in the municipality (*suburban*, n=7,286); and C which mostly includes areas located centrally in the municipality (*urban*, n=128,247). Mothers whose addresses were not classified into A, B, or C or had had no data on area of residence were categorised as not classified (n=284).^1, 2^

References:

Statistics Sweden, 2018. DeSo – demographic statistics areas (Swedish: *Demografiska statistikområden*) https://www.scb.se/hitta-statistik/regional-statistik-och-kartor/regionala-indelningar/deso---demografiska-statistikomraden/ cited 24 September 2024.

1. Kanamori M, Kondo N, Juarez S, Dunlavy A, Cederström A, Rostila M. Rural life and suicide: Does the effect of the community context vary by country of birth? A Swedish registry-based multilevel cohort study. Soc Sci Med. 2020 May; 253:112958.

**Appendix 3**

| **Table A3. The average marginal effects (AME) with 95% confidence intervals from binary logistic regression models exploring the association between any health condition; mental disorders; musculoskeletal conditions; and other health conditions; and eligibility for earnings-related parental leave benefits (N=151 452).** | | | | | | | |
| --- | --- | --- | --- | --- | --- | --- | --- |
|  | **Any healthcare treatment** | | **Specialist outpatient Care** | | **Hospitalisation** | | |
|  | **Model 1**  **AME (95% CI)** | **Model 2**  **AME (95% CI)** | **Model 1**  **AME (95% CI)** | **Model 2**  **AME (95% CI)** | **Model 1**  **AME (95% CI)** | | **Model 2**  **AME (95% CI)** |
| **Health in the year prior to pregnancy** | | | | | | |  |
| No health condition (reference) |  |  |  |  |  | |  |
| Any health condition | -0.013  (-0.015, -0.010) *** | -0.0068  (-0.0092, -0.0045) *** | -0.012  (-0.015, -0.0098) *** | -0.0065  (-0.0088, -0.0042) *** | -0.027  (-0.032, -0.023) *** | -0.018  (-0.022, -0.013) *** | |
|  |  |  |  |  |  |  | |
| No mental disorders (reference) |  |  |  |  |  |  | |
| Any mental disorders | -0.082  (-0.087, -0.078) *** | -0.066  (-0.071, -0.062) *** | -0.081  (-0.085, -0.076) *** | -0.065  (-0.069, -0.060) *** | -0.12  (-0.13, -0.11) *** | -0.087  (-0.098, -0.076) *** | |
|  |  |  |  |  |  |  | |
| No musculoskeletal condition (reference) |  |  |  |  |  |  | |
| Any musculoskeletal condition | 0.0047  (-0.0023, 0.012) | 0.0087  (0.0012, 0.015) * | 0.0047  (-0.0023, 0.012) | 0.0087  (0.0018, 0.016) * | -0.0038  (-0.031, 0.024) | 0.0032  (-0.024, 0.030) | |
|  |  |  |  |  |  |  | |
| No other health condition (reference) |  |  |  |  |  |  | |
| Any other health condition | -0.0085  (-0.011, -0.0061) *** | -0.0035  (-0.0058, -0.0011) ** | -0.0079  (-0.010, -0.0055) *** | -0.0030  (-0.0054, -0.00063) * | -0.020  (-0.025, -0.015) *** | -0.012  (-0.016, -0.007) *** | |
| **Health in the two consecutive years prior to pregnancy (chronic health)** | | | | | | | |
| No health condition (reference) |  |  |  |  |  | |  |
| Any health condition | -0.024  (-0.026, -0.021) *** | -0.018  (-0.018, -0.013) *** | -0.023  (-0.025, -0.020) *** | -0.015  (-0.017, -0.012) *** | -0.076  (-0.076, -0.058) *** | | -0.047  (-0.055, -0.038) *** |
|  |  |  |  |  |  | |  |
| No mental disorders (reference) |  |  |  |  |  | |  |
| Any mental disorders | -0.098  (-0.10, -0.092) *** | -0.079  (-0.085, -0.073) *** | -0.096  (-0.10, -0.091) *** | -0.078  (-0.084, -0.072) *** | -0.15  (-0.17, -0.13) *** | | -0.11  (-0.13, -0.92) |
|  |  |  |  |  |  | |  |
| No musculoskeletal condition (reference) |  |  |  |  |  | |  |
| Any musculoskeletal condition | -0.0050  (-0.016, 0.0064) | -0.00061  (-0.012, 0.011) | -0.0047  (-0.016, 0.0069) | -0.00041  (-0.012, 0.011) | § | | § |
|  |  |  |  |  |  | |  |
| No other health condition (reference) |  |  |  |  |  | |  |
| Any other health condition | -0.017  (-0.020, -0.014) *** | -0.0096  (-0.012, -0.0086) *** | -0.016  (-0.018, -0.013) *** | -0.0085  (-0.011, -0.0057) *** | -0.051  (-0.061, -0.040) *** | | -0.034  (-0.045, -0.023) *** |
| AME: average marginal effect; 95%CI: 95% confidence intervals, *p<0·05; **p<0·01; ***p<0·001; § Not enough units in cell to perform analysis for hospitalisation  Model 1: Adjusted for child’s year of birth mother’s age.  Model 2: Adjusted for child’s year of birth mother’s age, education. | | | | | | | |

**Appendix 4**

| **Table A4. The chronic health effects of any health condition, mental disorders, musculoskeletal conditions, and other health conditions in the year prior to pregnancy, and eligibility for earnings-related parental leave benefits by region of origin (n=151,452).** | | | | | | | | | | | | |
| --- | --- | --- | --- | --- | --- | --- | --- | --- | --- | --- | --- | --- |
|  | **Born in Sweden (n=133,527)** | | | | **Born in OECD regions (n=8,451)** | | | | **Born in non-OECD regions (n=9,474)** | | | |
|  | **N** | **%** | **Model 1**  **OR (95% CI)** | **Model 2**  **OR (95% CI)** | **N** | **%** | **Model 1**  **OR (95% CI)** | **Model 2**  **OR (95% CI)** | **N** | **%** | **Model 1**  **OR (95% CI)** | **Model 2**  **OR (95% CI)** |
| **Any health condition** |  |  |  |  |  |  |  |  |  |  |  |  |
| Any healthcare treatment | 104 632 | 78·36 | 1 |  | 6 505 | 76·97 | 1 | 1 | 6 888 | 72·70 | 1 | 1 |
| No healthcare treatment | 28 895 | 21·64 | 0·64  (0·61-0·68) *** | 0·74  (0·70-0·78) *** | 1 946 | 23·03 | 0·76  (0·65-0·89) ** | 0·87  (0·74-1·03) | 2 586 | 27·30 | 0·76  (0·68-0·85) *** | 0·81  (0·72-0·90) *** |
|  |  |  |  |  |  |  |  |  |  |  |  |  |
| No outpatient care | 105 411 | 78·94 | 1 |  | 6 549 | 77·49 | 1 | 1 | 6 965 | 73·52 | 1 | 1 |
| Outpatient care | 28 116 | 21·06 | 0·65  (0·62-0·69) *** | 0·75  (0·71-0·80) *** | 1 902 | 22·51 | 0·77  (0·65-0·90) ** | 0·88  (0·74-1·04) | 2 509 | 26·48 | 0·79  (0·71-0·88) *** | 0·84  (0·75-0·94) ** |
|  |  |  |  |  |  |  |  |  |  |  |  |  |
| No hospitalisation | 132 609 | 99·31 | 1 |  | 8 400 | 99·40 | 1 | 1 | 9 363 | 98·83 | 1 | 1 |
| Hospitalisation | 918 | 0·69 | 0·27  (0·22-0·32) *** | 0·37  (0·30-0·45) *** | 51 | 0·60 | 0·32  (0·17-0·61) ** | 0·49  (0·25-0·96) * | 111 | 1·17 | 0·55  (0·36-0·82) ** | 0·64  (0·42-0·98) * |
|  |  |  |  |  |  |  |  |  |  |  |  |  |
| **Mental disorders** |  |  |  |  |  |  |  |  |  |  |  |  |
| Any healthcare treatment | 131 796 | 98·70 | 1 |  | 8 364 | 98·97 | 1 | 1 | 9 363 | 98·83 | 1 | 1 |
| No healthcare treatment | 1 731 | 1·30 | 0·13  (0·12-0·15) *** | 0·18  (0·16-0·20) *** | 87 | 1·03 | 0·25  (0·16-0·40) *** | 0·33  (0·20-0·54) *** | 111 | 1·17 | 0·33  (0·22-0·48) *** | 0·38  (0·26-0·57) *** |
|  |  |  |  |  |  |  |  |  |  |  |  |  |
| No outpatient care | 131 868 | 98·76 | 1 |  | 8 369 | 99·03 | 1 | 1 | 9 372 | 98·92 | 1 | 1 |
| Outpatient care | 1 659 | 1·24 | 0·13  (0·12-0·15) *** | 0·18  (0·16-0·20) *** | 82 | 0·97 | 0·27  (0·17-0·45) *** | 0·35  (0·21-0·58) *** | 102 | 1·08 | 0·37  (0·25-0·56) *** | 0·44  (0·29-0·66) *** |
|  |  |  |  |  |  |  |  |  |  |  |  |  |
| No hospitalisation | 133 422 | 99·92 | 1 |  | 8 445 | 99·93 | § | § | 9 468 | 99·94 | § | § |
| Hospitalisation | 105 | 0·08 | 0·05  (0·04-0·08) *** | 0·10  (0·06-0·15) *** | 6 | 0·07 |  |  | 6 | 0·06 |  |  |
|  |  |  |  |  |  |  |  |  |  |  |  |  |
| **Musculoskeletal conditions** |  |  |  |  |  |  |  |  |  |  |  |  |
| Any healthcare treatment | 132 131 | 98·95 | 1 |  | 8 386 | 99·23 | 1 | 1 | 9 385 | 99·06 | 1 | 1 |
| No healthcare treatment | 1 396 | 1·05 | 0·88  (0·69-1·13) | 0·96  (0·75-1·24) | 65 | 0·77 | 0·78  (0·37-1·64) | 0·91  (0·42-1·94) | 89 | 0·94 | 0·90  (0·54-1·50) | 0·93  (0·55-1·58) |
|  |  |  |  |  |  |  |  |  |  |  |  |  |
| No outpatient care | 132 165 | 98·98 | 1 |  | 8 387 | 99·24 | 1 | 1 | 9 392 | 99·13 | 1 | 1 |
| Outpatient care | 1 362 | 1·02 | 0·87  (0·68-1·12) | 0 ·92  (0·71-1·19) | 64 | 0·76 | 0·77  (0·36-1·62) | 0·92  (0·42-2·02) | 82 | 0·87 | 0·93  (0·54-1·59) | 0·78  (0·45-1·38) |
|  |  |  |  |  |  |  |  |  |  |  |  |  |
| No hospitalisation | 133 503 | 99·98 | § | § | 8 450 | 99·99 | § | § | 9 470 | 99·96 | § | § |
| Hospitalisation | 24 | 0·02 |  |  | 1 | 0·01 |  |  | 4 | 0·04 |  |  |
|  |  |  |  |  |  |  |  |  |  |  |  |  |
| **Other health conditions** |  |  |  |  |  |  |  |  |  |  |  |  |
| Any healthcare treatment | 107 905 | 80·81 | 1 |  | 6 668 | 78·90 | 1 | 1 | 7 099 | 74·93 | 1 | 1 |
| No healthcare treatment | 25 622 | 19·19 | 0·77  (0·72-0·82) *** | 0·87  (0·82-0·93) *** | 1 783 | 21·10 | 0·80  (0·68-0·95) * | 0·92  (0·77-1·09) | 2 375 | 25·07 | 0·79  (0·70-0·88) *** | 0·83  (0·74-0·93) ** |
|  |  |  |  |  |  |  |  |  |  |  |  |  |

| No outpatient care | 108 677 | 81·39 | 1 |  | 6 716 | 79·47 | 1 | 1 | 7 176 | 75·74 | 1 | 1 |
| --- | --- | --- | --- | --- | --- | --- | --- | --- | --- | --- | --- | --- |
| Outpatient care | 24 850 | 18·61 | 0·79  (0·74-0·84) *** | 0·89  (0·84-0·95) ** | 1 735 | 20·53 | 0·80  (0·68-0·95) * | 0·92  (0·77-1·09) | 2 298 | 24·26 | 0·81  (0·72-0·91) *** | 0·85  (0·76-0·96) ** |
|  |  |  |  |  |  |  |  |  |  |  |  |  |
| No hospitalisation | 132 804 | 99·46 | 1 |  | 8 408 | 99·49 | 1 | 1 | 9 377 | 98·98 | 1 | 1 |
| Hospitalisation | 723 | 0·54 | 0·40  (0·31-0·51) *** | 0·53  (0·41-0·68) *** | 43 | 0·51 | 0·36  (0·18-0·74) ** | 0·53  (0·23-1·12) | 97 | 1·02 | 0·59  (0·38-0·93) * | 0·68  (0·43-1·07) |
| OR: odds ratio; 95%CI: 95% confidence intervals.  Model 1: Adjusted for child’s year of birth, mother’s age  Model 2: Adjusted for child’s year of birth, mother’s age, education  *p<0·05; **p<0·01; ***p<0·001.  § Not enough units in cell to perform analyse. | | | | | | | | | | | | |

**Appendix 5**

| **Table A5. The average marginal effects (AME) with 95% confidence intervals from binary logistic regression models exploring the association between any health condition; mental disorders; musculoskeletal conditions; and other health conditions; and eligibility for earnings-related parental leave benefits by region of origin (N=151 452).** | | | | | | |
| --- | --- | --- | --- | --- | --- | --- |
|  | **Born in Sweden (n=133 527)** | | **Born in OECD regions (n=8 451)** | | **Born in non-OECD regions (n=9 474)** | |
|  | **Model 1**  **AME (95% CI)** | **Model 2**  **AME (95% CI)** | **Model 1**  **AME (95% CI)** | **Model 2**  **AME (95% CI)** | **Model 1**  **AME (95% CI)** | **Model 2**  **AME (95% CI)** |
| **Any health condition** |  |  |  |  |  |  |
| No healthcare treatment (reference) |  |  |  |  |  |  |
| Any healthcare treatment | -0.0096  (-0.012, -0.0073) *** | -0.0052  (-0.0075, -0.0030) *** | -0.013  (-0.026, -0.00019) * | -0.0052  (-0.018, 0.0076) | -0.016  (-0.032, 0.00056) | -0.0060  (-0.022, 0.0099) |
|  |  |  |  |  |  |  |
| No outpatient care (reference) |  |  |  |  |  |  |
| Outpatient care | -0.0093  (-0.012, -0.0071) *** | -0.0051  (-0.0072, -0.0028) *** | -0.013  (-0.026, 0.00038) | -0.0045  (-0.017, 0.0084) | -0.013  (-0.029, 0.0031) | -0.0044  (-0.020, 0.011) |
|  |  |  |  |  |  |  |
| No hospitalisation (reference) |  |  |  |  |  |  |
| Hospitalisation | -0.023  (-0.027, -0.018) *** | -0.015  (-0.019, -0.011) *** | -0.035  (-0.059, -0.010) ** | -0.023  (-0.048, 0.001) | -0.039  (-0.069, -0.00) * | -0.024  (-0.0529, 0.005) |
|  |  |  |  |  |  |  |
| **Mental disorders** |  |  |  |  |  |  |
| No healthcare treatment (reference) |  |  |  |  |  |  |
| Any healthcare treatment | -0.073  (-0.077, -0.069) *** | -0.060  (-0.064, -0.056) *** | -0.090  (-0.12, -0.057) *** | -0.070  (-0.10, -0.037) *** | -0.15  (-0.19, -0.11) *** | -0.13  (-0.17, -0.86) *** |
|  |  |  |  |  |  |  |
| No outpatient care (reference) |  |  |  |  |  |  |
| Outpatient care | -0.072  (-0.076, -0.068) *** | -0.059  (-0.064, -0.055) *** | -0.078  (0.011, -0.043) *** | -0.060  (-094, -0.025) ** | -0.14  (-0.18, -0.096) *** | -0.12  (-0.16, -0.078) *** |
|  |  |  |  |  |  |  |
| No hospitalisation (reference) |  |  |  |  |  |  |
| Hospitalisation | -0.099  (-0.11, -0.089) *** | -0.076  (-0.086, -0.067) *** | -0.15  (-0.22, -0.073) *** | -0.12  (-0.20, -0.044) ** | -0.22  (-0.32, -0.12) *** | -0.16  (-0.26, -0.062) ** |
|  |  |  |  |  |  |  |
| **Musculoskeletal conditions** |  |  |  |  |  |  |
| No healthcare treatment (reference) |  |  |  |  |  |  |
| Any healthcare treatment | -0.000042  (-0.0064, 0.0063) | 0.0033  (-0.0030, 0.0096) | 0.0012  (-0.038, 0.040) | 0.015  (-0.024, 0.053) | 0.073  (0.020, 0.13) ** | 0.070  (0.020, 0.12) ** |
|  |  |  |  |  |  |  |
| No outpatient care (reference) |  |  |  |  |  |  |
| Outpatient care | -0.00021  (-0.0066, 0.0062) | 0.0031  (-0.0032, 0.0094) | 0.00056  (-0.038, 0.040) | 0.014  (-0.025, 0.053) | 0.078  (0.024, 0.13) ** | 0.076  (0.024, 0.13) ** |
|  |  |  |  |  |  |  |
| No hospitalisation (reference) |  |  |  |  |  |  |
| Hospitalisation | -0.0029  (-0.029, 0.023) | 0.0035  (-0.022, -0.029) | 0.0072  (-0.18, 0.19) | 0.025  (-0.16, 0.21) | -0.058  (-0.24, 0.13) | -0.060  (-0.24, 0.12) |
|  |  |  |  |  |  |  |
| **Other health conditions** |  |  |  |  |  |  |
| No healthcare treatment (reference) |  |  |  |  |  |  |
| Any healthcare treatment | -0.0049  (-0.0072, -0.0026) *** | -0.0012  (-0.0034, 0.0011) | -0.011  -0.025, 0.0016 | -0.0039  (-0.17, 0.009) | -0.015  (-0.031, 0.0017) | -0.0052  (-0.021, 0.011) |
|  |  |  |  |  |  |  |
| No outpatient care (reference) |  |  |  |  |  |  |
| Outpatient care | -0.0045  (-0.0067, -0.0022) *** | -0.00085  (-0.0031, 0.0014) | -0.011  (-0.024, 0.0025) | -0.0029  (-0.016, 0.010) | -0.012  (-0.028, 0.0047) | -0.0034  (-0.019, 0.013) |
|  |  |  |  |  |  |  |
| No hospitalisation (reference) |  |  |  |  |  |  |
| Hospitalisation | -0.014  (-0.019, -0.010) *** | -0.0082  (-0.013, -0.0036) *** | -0.031  (-0.057, -0.006) * | -0.020  (-0.045, 0.0056) | -0.032  (-0.063, -0.00074) * | -0.019  (0.050, 0.011) |
| 95%CI: 95% confidence intervals, *p<0·05; **p<0·01; ***p<0·001  Model 1: Adjusted for child’s year of birth mother’s age.  Model 2: Adjusted for child’s year of birth mother’s age, education. | | | | | | |

**Appendix 6**

| **Table A6. Sensitivity analysis: the association between specialist outpatient care for any health condition, mental disorders, musculoskeletal conditions and other health conditions, and eligibility for earnings-related parental leave benefits (n=151 452).** | | | | | | | | |
| --- | --- | --- | --- | --- | --- | --- | --- | --- |
|  | **Health in the year prior to pregnancy** | | | | **Health in the two consecutive year prior to pregnancy (Chronic health)** | | | |
|  | **N** | **%** | **Model 1**  **OR (95% CI)** | **Model 2**  **OR (95% CI)** | **N** | **%** | **Model 1**  **OR (95% CI)** | **Model 2**  **OR (95% CI)** |
| No health condition | 96 149 | 63·48 | 1 | 1 | 126 145 | 83·29 | 1 | 1 |
| Any health condition | 55 303 | 36·52 | 0·89 (0·86-0·94) *** | 0·96 (0·92-1·00) | 25 307 | 16·71 | 0·82 (0·77-0·87) *** | 0·90 (0·85-0·95) *** |
|  |  |  |  |  |  |  |  |  |
| No mental disorder | 148 081 | 97·77 | 1 | 1 | 149 952 | 99·01 | 1 | 1 |
| Any mental disorder | 3 371 | 2·23 | 0·25 (0·23-0·27) *** | 0·31 (0·28-0·34) *** | 1 500 | 0·99 | 0·20 (0·17-0·22) *** | 0·25 (0·22-0·28) *** |
|  |  |  |  |  |  |  |  |  |
| No musculoskeletal condition | 146 836 | 96·95 | 1 | 1 | 150 152 | 99·14 | 1 | 1 |
| Any musculoskeletal condition | 4 616 | 3·05 | 1·10 (0·96-1·26) | 1·19 (1·04-1·36) * | 1 300 | 0·86 | 1·09 (0·80-1·30) | 1·08 (0·85-1·39) |
|  |  |  |  |  |  |  |  |  |
| No other health condition | 99 082 | 65·42 | 1 | 1 | 128 700 | 84·98 | 1 | 1 |
| Any other health condition | 52 370 | 34·58 | 0·92 (0·88-0·97) ** | 0·98 (0·94-1·03) | 22 752 | 15·02 | 0·88 (0·83 -0·94) *** | 0·96 (0·90-1·02) |
| OR: odds ratio; 95%CI: 95% confidence intervals.  Model 1: Adjusted for child’s year of birth, mother’s age  Model 2: Adjusted for child’s year of birth, mother’s age, education  **p<0·01; ***p<0·001 | | | | | | | | |
